# Horizontal Tension-Band Plating: A New Strategy for Displaced and Multifragmentary Anterior Tibial Tuberosity Fractures Associated with Complex Metaphyseal Fractures

**DOI:** 10.1055/s-0046-1822832

**Published:** 2026-07-14

**Authors:** Robinson Esteves Pires, Gustavo Waldolato Silva, Rafael Chagas Silva, Erick Veiga Franco da Rosa

**Affiliations:** 1 Orthopedics and Traumatology Service, Hospital Felício Rocho, Belo Horizonte, MG, Brazil; 2 Locomotor System Department, School of Medicine, Universidade Federal de Minas Gerais, Belo Horizonte, MG, Brazil

**Keywords:** knee fractures, tibia, tibial plateau fractures, fraturas do joelho, fraturas do planalto tibial, tíbia

## Abstract

There is no standardized treatment for multifragmentary fractures of the anterior tibial tuberosity (ATT) in adult patients, with or without concomitant metaphyseal fractures of the proximal tibia. This technical note aims to demonstrate the results of using a minifragment plate positioned horizontally according to the tension band principle in two patients with multifragmentary fractures of the ATT.

## Resumo

Não existe padronização de tratamento para as fraturas multifragmentares da tuberosidade anterior da tíbia (TAT) nos pacientes adultos, com ou sem associação com fraturas metafisárias da tíbia proximal. O objetivo desta nota técnica é demonstrar os resultados da utilização da placa de minifragmentos colocada segundo o princípio da banda de tensão, de forma horizontal, em dois pacientes portadores de fratura multifragmentar da TAT.

## Introduction

High-energy proximal tibial fractures involving the anterior tibial tuberosity (ATT) represent a significant challenge in orthopedics due to their biomechanical complexity. These injuries often result from high-energy trauma and lead to multifragmentary fractures with extensive soft tissue involvement, further complicating clinical and surgical management. 1 2 3 As the attachment site for the knee extensor mechanism, the ATT is subjected to intense biomechanical forces during muscle contraction. Due to the constant action of the extensor mechanism and the forces it generates, fractures involving the ATT—especially those that are displaced or multifragmentary—carry a high risk of fixation failure and may compromise fracture healing and functional recovery. 1 2 3

The current literature is inconsistent regarding the optimal method for treating displaced ATT fractures. Treatment options include fixation with lag screws, either cannulated or noncannulated; combinations of plates and screws; conventional tension-band techniques; or reattachment of the patellar ligament to the tibia using anchors. 4 5 However, there is no clear consensus on which approach offers greater mechanical stability and superior clinical outcomes, particularly in multifragmentary fractures, for which fixation with conventional methods is not feasible.

This technical note aims to analyze the effectiveness of surgical fixation using a minifragment plate positioned horizontally according to the tension band principle to maintain reduction and promote fracture healing in displaced ATT fractures in adult patients.

## Materials and Methods

This study included two adult patients with proximal metaphyseal tibial fractures characterized by ATT displacement and/or fragmentation. Cases were selected retrospectively based on medical records, outpatient clinical examinations, and radiographic findings. The study included patients from a tertiary hospital who underwent surgical fixation with a minifragment plate positioned horizontally according to the tension band principle. Follow-up continued until clinical and radiographic fracture healing (maximum of 1 year), assessed using serial conventional radiographs.

Ethical approval for article publication was granted under the CAAE 93952425.0.0000.5125.

### Case Presentation

#### Patient 1

A 38-year-old male patient, a victim of a motorcycle accident, presented with an open fracture of the proximal region of the left leg, classified as Gustilo-Anderson type IIIA. At admission, two cutaneous wounds with bone exposure were observed at the fracture site, with no clinical signs of neurovascular compromise. The patient underwent urgent surgical management with debridement of devitalized tissues and transarticular external fixation for initial stabilization and local damage control.

After 3 days, a second surgical procedure was performed, including further debridement, removal of the external fixator, and conversion to definitive internal fixation. The fixation strategy combined two bridge plates (medial and lateral) for the metaphyseal component of the proximal tibia, minifragment plates for fragment-specific fixation, and a plate positioned horizontally for the multifragmentary ATT fracture. Because of the degree of comminution, the minifragment plate was passed within the patellar ligament (transtendon approach) to achieve greater fixation stability.

**Fig. 1:**
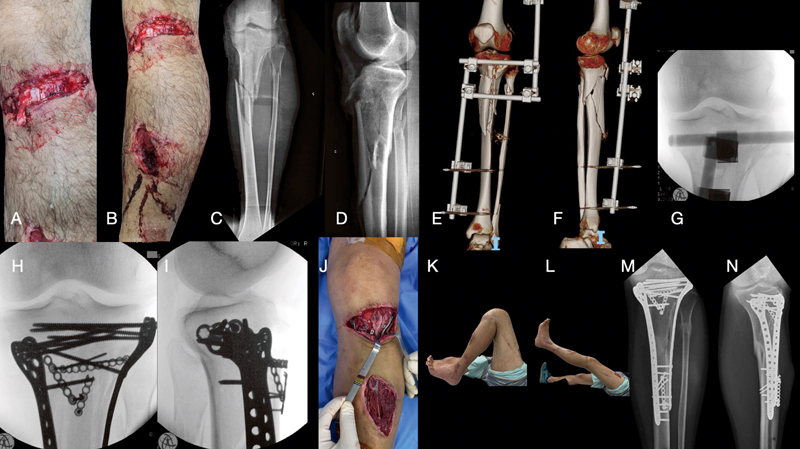
First patient. ( **A** , **B** ) Preoperative photographs demonstrating wounds in the anterior region of the left leg with exposure of the anterior tibial tuberosity (ATT). ( **C** , **D** ) Radiographs of the left leg in anteroposterior (AP) and lateral views demonstrating a proximal tibial fracture with a high degree of multifragmentation and diaphyseal extension. ( **E** , **F** ) Computed tomography (CT) with three-dimensional (3D) reconstruction demonstrating fragmentation of the ATT. ( **G** ) Fluoroscopic image of the left knee in the AP view demonstrating application of a transarticular external fixator. ( **H** , **I** ) Intraoperative images demonstrating anatomical reduction of the articular surface with fixation using 2.4-mm Evos Mini Plating System minifragment plates and two 3.5-mm Evos Plating System bridge plates in the medial and lateral columns of the proximal tibia (Smith & Nephew). ( **J** ) A plate positioned horizontally according to the tension band principle is demonstrated, passed within the patellar ligament at its insertion into the ATT to contain the multifragmentary fracture. ( **K** , **L** ) Postoperative photographs demonstrating adequate soft tissue healing and functional recovery of the knee, with complete restoration of range of motion. ( **M** , **N** ) Radiographs in AP and lateral views, demonstrating complete fracture healing, maintenance of reduction of the ATT, and no signs of fixation failure.

In the postoperative period, the distal leg wound was managed with negative pressure therapy, followed by skin grafting for definitive soft tissue coverage. Fig. 1 illustrates the initial trauma and surgical treatment stages in patient 1.

The tension-band plate was removed after complete healing of the fracture (17 months after fixation) to minimize the risk of additional injury from friction with the patellar ligament.

#### Patient 2

**Fig. 2:**
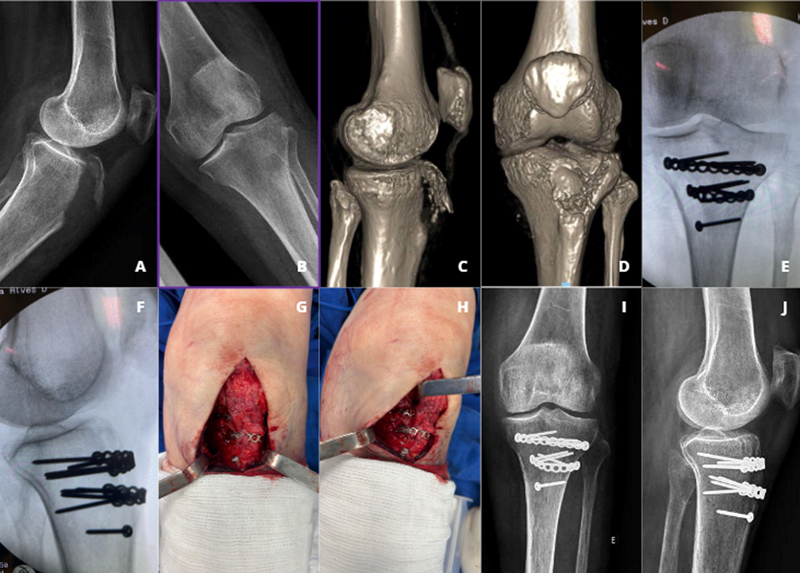
Second patient. ( **A** , **B** ) Radiographs of the left knee in anteroposterior (AP) and lateral views, demonstrating a displaced fracture of the anterior tibial tuberosity (ATT). ( **C** , **D** ) Computed tomography scans with three-dimensional (3D) reconstruction demonstrating displacement and multifragmentation of the ATT. ( **E** , **F** ) Fluoroscopic images in anteroposterior and lateral views demonstrating fixation with a minifragment plate positioned horizontally according to the tension band principle. ( **G** , **H** ) Intraoperative photographs demonstrating the surgical approach and the final plate configuration. ( **I** , **J** ) Follow-up radiographs demonstrating maintenance of reduction, absence of implant failure, and satisfactory fracture healing.

The second case refers to an 82-year-old female patient, with Alzheimer's disease and osteoporosis, who was previously ambulatory with the aid of a cane, although with functional limitations. The patient sustained a fall with direct trauma to the left knee, resulting in ATT displacement and multifragmentation. A definitive surgical treatment with open reduction and internal fixation of the ATT, using 2 minifragment plates of 2.4 mm (Evos Mini Plating System, Smith & Nephew) positioned according to the tension band principle, conferring adequate stability and maintain reduction, as evidenced by fluoroscopic intraoperative evaluation. This case did not require transtendon passage of the plates through the patellar ligament. Fig. 2 illustrates the initial trauma and surgical treatment stages in patient 2.

As with patient 1, both plates were removed after fracture healing (3 months after fixation) to avoid additional injury from friction with the patellar ligament.

## Discussion

The available literature on the effectiveness and short- and long-term outcomes of different treatment methods for displaced and/or multifragmentary ATT fractures in adult patients is limited, consisting predominantly of case reports and small clinical series, without comparative studies or higher-level evidence. 4 5 Limited data make it difficult to standardize management of these injuries and highlight the need for alternative strategies, capable of providing adequate stability, maintaining reduction, and promoting fracture healing in biomechanically challenging fractures. Using plates positioned horizontally in proximal tibial fractures is not a new concept.

Horizontal or semicircular plate configurations (barrel, hoop, or rim plates) have been proposed to treat complex proximal tibial fractures. 4 5 6 7 Giordano et al. 6 described the hoop plate as a method to contain the posterior perimeter in tibial plateau fractures, serving as a peripheral stabilizing element for posterior fragments. Similarly, Rojas et al. 7 described the umbrella technique for complex proximal tibial fractures, combining an anterior hoop plate with an intramedullary nail to increase construct stability and contain the anterior perimeter during nail insertion.

Our technique follows a distinct biomechanical principle, functioning as a true tension band that “embraces” the multifragmentary ATT fracture and promotes dynamic containment of the fragments, even during knee flexion. The *tension band* concept is based on a classic biomechanical principle described by the AO Foundation, in which an implant placed on the tension side of a bone converts tensile forces into compressive forces at the fracture site during functional mobilization. In ATT fractures, knee flexion generates traction from the extensor mechanism, producing tensile forces at the proximal anterior cortex of the tibia. When a plate is positioned horizontally over the ATT, it acts as a tension-band device, neutralizing these forces and promoting dynamic compression at the fracture site during quadriceps contraction and knee motion.

This configuration may involve one or two plates, depending on fragment size and fixation requirements. Due to its low profile (2.0 or 2.4 mm), the plate may, when necessary, pass through the patellar ligament without significantly compromising the soft tissues, thereby expanding the technical possibilities in complex scenarios. To the best of our knowledge, no previous reports describe the use of horizontally positioned plates according to the tension band principle for fixation of ATT fractures.

This study has inherent design limitations. This is a technical note evaluating the efficacy of an alternative fixation method in only two patients, lacking a comparative group treated with conventional techniques. Long-term evaluation was not performed, and no functional scores or validated quality-of-life instruments were used. The small sample size also precludes an inferential statistical analysis, limiting interpretation to a descriptive analysis. In both cases herein reported, fixation with a horizontally-positioned plate following the tension band principle maintained reduction, provided adequate stability, and enabled fracture healing with no evidence of implant failure. These initial findings suggest that the technique may be a viable option in the therapeutic armamentarium and support the need for future studies with larger samples, longer follow-up, and comparative functional assessments to better define its indications and clinical outcomes.

## Final Considerations

Fixation with a minifragment plate positioned horizontally according to the tension band principle proved to be a safe and effective alternative for treating complex and displaced ATT fractures in the two patients evaluated. The technique provided adequate mechanical stability to maintain reduction throughout follow-up, allowing satisfactory fracture healing.
